# OCT particle tracking velocimetry of biofluids in a microparallel plate strain induction chamber

**DOI:** 10.1117/1.JBO.26.9.096005

**Published:** 2021-09-15

**Authors:** Kelsey J. Oeler, David B. Hill, Amy L. Oldenburg

**Affiliations:** a University of North Carolina at Chapel Hill, Department of Biomedical Engineering, Chapel Hill, North Carolina, United States; b University of North Carolina at Chapel Hill, Department of Physics and Astronomy, Marsico Lung Institute, Chapel Hill, North Carolina, United States; c University of North Carolina at Chapel Hill, Department of Physics and Astronomy, Chapel Hill, North Carolina, United States

**Keywords:** optical coherence tomography, mucus, motion tracking, particle tracking velocimetry, rheometry

## Abstract

**Significance:** Imaging biofluid flow under physiologic conditions aids in understanding disease processes and health complications. We present a method employing a microparallel plate strain induction chamber (MPPSIC) amenable to optical coherence tomography to track depth-resolved lateral displacement in fluids in real time while under constant and sinusoidal shear.

**Aim:** Our objective is to track biofluid motion under shearing conditions found in the respiratory epithelium, first validating methods in Newtonian fluids and subsequently assessing the capability of motion-tracking in bronchial mucus.

**Approach:** The motion of polystyrene microspheres in aqueous glycerol is tracked under constant and sinusoidal applied shear rates in the MPPSIC and is compared with theory. Then 1.5 wt. % bronchial mucus samples considered to be in a normal hydrated state are studied under sinusoidal shear rates of amplitudes 0.7 to $3.2\;s^{-1}$.

**Results:** Newtonian fluids under low Reynolds conditions (Re∼10−4) exhibit velocity decreases directly proportional to the distance from the plate driven at both constant and oscillating velocities, consistent with Navier–Stokes’s first and second problems at finite depths. A 1.5 wt. % mucus sample also exhibits a uniform shear strain profile.

**Conclusions:** The MPPSIC provides a new capability for studying biofluids, such as mucus, to assess potentially non-linear or strain-rate-dependent properties in a regime that is relevant to the mucus layer in the lung epithelium.

## 1 Introduction

Biofluids are composed of macromolecules that exhibit complex and spatially heterogeneous viscoelastic properties. In disease states, these viscoelastic properties can become altered, changing the way a biofluid moves and potentially exacerbating effects of the disease, such as occurs for airway surface liquid in pulmonary disease.^1^^,^^2^ For a proper understanding of how disease states modify biofluid flow, it is necessary to quantify fluid flow *in situ* under physiological conditions over a wide field of view, allowing one to characterize the system over its full span (such as the thickness of the airway surface layer) and to detect heterogeneity in the fluid flow arising from intrinsic viscoelastic heterogeneity or from heterogeneous applied strain. However, the ability to investigate fluid flow under these conditions is technically challenging. There have been many advances toward visualizing and quantifying rheological behaviors by tracking particles embedded in the fluid with a variety of imaging modalities. Current methods for particle tracking in holography and microscopy are limited in their ability to simultaneously provide a wide field of view (laterally and in depth) and real-time imaging while maintaining microscopic resolution.^3^^–^^5^ Optical coherence tomography (OCT) is emerging as a modality that is particularly amenable to characterizing fluid flow and rheological properties because it offers high-speed, depth-resolved imaging with microscopic resolution.^6^^,^^7^ Here we propose a new platform for visualizing biofluid motion under physiologic shear with OCT, which is capable of tracking microscale fluid motions over millimeter fields of view, in combination with a microparallel plate strain induction chamber (MPPSIC) amenable to real-time OCT imaging.

As a first demonstration, the MPPSIC is tailored to recapitulate the lung epithelium’s mucociliary clearance (MCC) system. MCC is the airway’s defense mechanism that entraps inhaled pathogens in mucus and expels the mucus with hair-like appendages called cilia.^8^^–^^10^ Cilia induce strain on mucus and the rheological response of the fluid is largely dictated by its polymeric mucin meshwork.^11^^,^^12^ Pulmonary diseases such as cystic fibrosis and chronic obstructive pulmonary disease are characterized by dehydrated (high wt. %) mucus that cilia are unable to propel out of the respiratory system. This breakdown of MCC causes accumulation of an increasingly hyperviscous mucus layer that weighs down the cilia and further hinders MCC in a vicious cycle.^13^^,^^14^ However, how this deleterious process is affected by the viscoelastic properties of mucus, which is known to be highly shear-thinning, as well as heterogeneity in mucus wt. %, is not yet well-understood.^15^ Diffusive nanoprobes for imaging mucus wt. % with OCT have begun to shed light on how the MCC is improved by the use of nebulized therapeutics,^16^ but this method does not provide direct information about mucus viscosity, i.e., how mucus responds to shear. Mucus is subjected to a range of shear strains by the cilia; how the strain propagates over the mucus layer thickness of ∼10 to 100  μm and affects the subsequent MCC is not known. OCT has been shown to be effective for visualizing mucus flow in both *in vitro* and *in vivo* experiments.^17^^,^^18^ Some studies used OCT to perform direct measurements of cilia-driven fluid flow using methods such as particle tracking velocimetry^19^^–^^21^ and OCT particle image velocimetry.^22^ Here we describe a method using OCT to track particle probes in a controlled MCC-mimicking environment, giving way for investigative studies over a wide range of strain rates.

In this paper, we present an MPPSIC designed to mimic the MCC. By designing a strain induction chamber amenable to OCT imaging, for the first time, we demonstrate depth-resolved visualization of fluid motion induced by controlled, dynamic shearing. Motion tracking is first validated using Newtonian fluids composed of glycerol–water mixtures under low Reynolds conditions such that the observed motion has a simple theoretical analytical solution for validation. Then a bronchial mucus sample at 1.5 wt. %, a concentration consistent with a healthy airway epithelium, was tested in the MPPSIC. In Sec. 2, we present the theoretical solution for Newtonian fluids under dynamic shear in a parallel plate strain induction chamber. In Sec. 3, we present the design of the chamber, the OCT system, and the experimental procedures, and in Sec. 4 we provide details of the motion-tracking image analysis. Results and discussion of our findings in Newtonian fluids and in mucus are given in Secs. 5 and 6, respectively. Our demonstrations show that the MPPSIC successfully tracks biofluid flow in conditions that recapitulate the airway surface liquid on the lung epithelium. As will be discussed below, this platform may also be amenable for quantifying fluid rheological properties in future implementations.

## 2 Theory

To validate the MPPSIC, we first write the analytical solutions for the depth-dependent fluid velocity under the conditions used in our experiments. Our validation experiments are performed with Newtonian fluids between parallel plates, whereas the bottom plate is displaced laterally with a periodic waveform, either triangular (to impart constant shear rate) or sinusoidal (to impart an oscillatory shear rate). In all experiments, the plates were measured to have an angle difference of <1  deg; thus we treat them as parallel here. Using OCT, we quantify the velocity of polystyrene microspheres added to the fluids for particle tracking. The microspheres are presumed to have no-slip and negligible Brownian motion such that their motion represents that of the surrounding fluid. The plates are much wider than the OCT lateral field of view, so we can neglect edge effects, and the bottom plate is driven along the x axis, so we may assume that fluid velocity is purely along the x axis, $V=V_{x}$ and only depends on the depth from the top plate z and time t. The fluid is assumed to follow a no-slip condition with both plates, and the plate separation is given as H. The transient time of the fluid depends on kinematic viscosity v. The transient time of the Newtonian fluids used in our experiments was calculated to be negligible at <1  ms.^23^ The Reynolds numbers of the Newtonian fluids were considered low at a maximum value of $1.3\times 10^{-4}$.

For experiments employing a constant shear rate, a triangle waveform drives the bottom plate of the MPPSIC at a constant velocity, $\pm V_{x,0}$ in upswing and downswing, respectively; for simplicity, we treat each direction of motion equally and ignore the sign of the velocity. This situation is equivalent to Navier–Stokes’s first problem for a finite depth field in which a Newtonian fluid is induced with a constant shear rate at one boundary. The steady-state solution when the Reynolds condition is low exhibits a constant velocity gradient in depth, such that^24^

$$V_{x}=V_{x}(z)=\frac{V_{x0}z}{H}.$$ (1)

In the steady state, the fluid velocity decreases with direct proportionality to the distance from the plate moving at velocity $V_{x,0}$. In this case, fluid velocity can be tracked at various known depths, and the shear rate $\dot{\gamma }$ is constant and given by the velocity gradient:

$$\dot{\gamma }=\frac{dV_{x}(z)}{dz}=\frac{V_{x0}}{H}.$$ (2)

To more closely mimic physiological conditions of the MCC, we then performed experiments under sinusoidal shear by driving the bottom plate with a sinusoidal waveform of angular frequency ω and velocity amplitude $U_{x,0}$. For a Newtonian fluid, the solution of the Navier–Stokes’s second problem for a finite depth field holds for sinusoidal conditions in the steady state. In this solution, a fluid induced with a sinusoidal shear under low Reynolds conditions again exhibits a velocity gradient constant in depth and follow the time dependence of the driving waveform such that^24^

$$U_{x}=\frac{U_{x0}z}{H}\cos(\omega t+\phi )=U_{x\max}(z)\cos(\omega t+\phi ),$$ (3)

and the phase, denoted as ϕ, is equal to that of the driving waveform (no phase lag under low Reynolds conditions). In this solution, the fluid velocity amplitude $U_{x,\max}(z)$ has the same form as the constant velocity versus depth in Eq. (1), which decreases with direct proportionality to the distance from the plate. By tracking $U_{x,\max}(z)$ at various known depths, the maximum shear rate (equivalent to the shear rate amplitude) in time is constant in depth and is extracted by the gradient of the velocity amplitude:

$${\dot{\gamma }}_{\max}=\frac{dU_{x\max}(z)}{dz}=\frac{U_{x0}}{H}.$$ (4)

## 3 Materials and Methods

### 3.1 Microparallel Plate Strain Induction Chamber

**Fig. 1:**
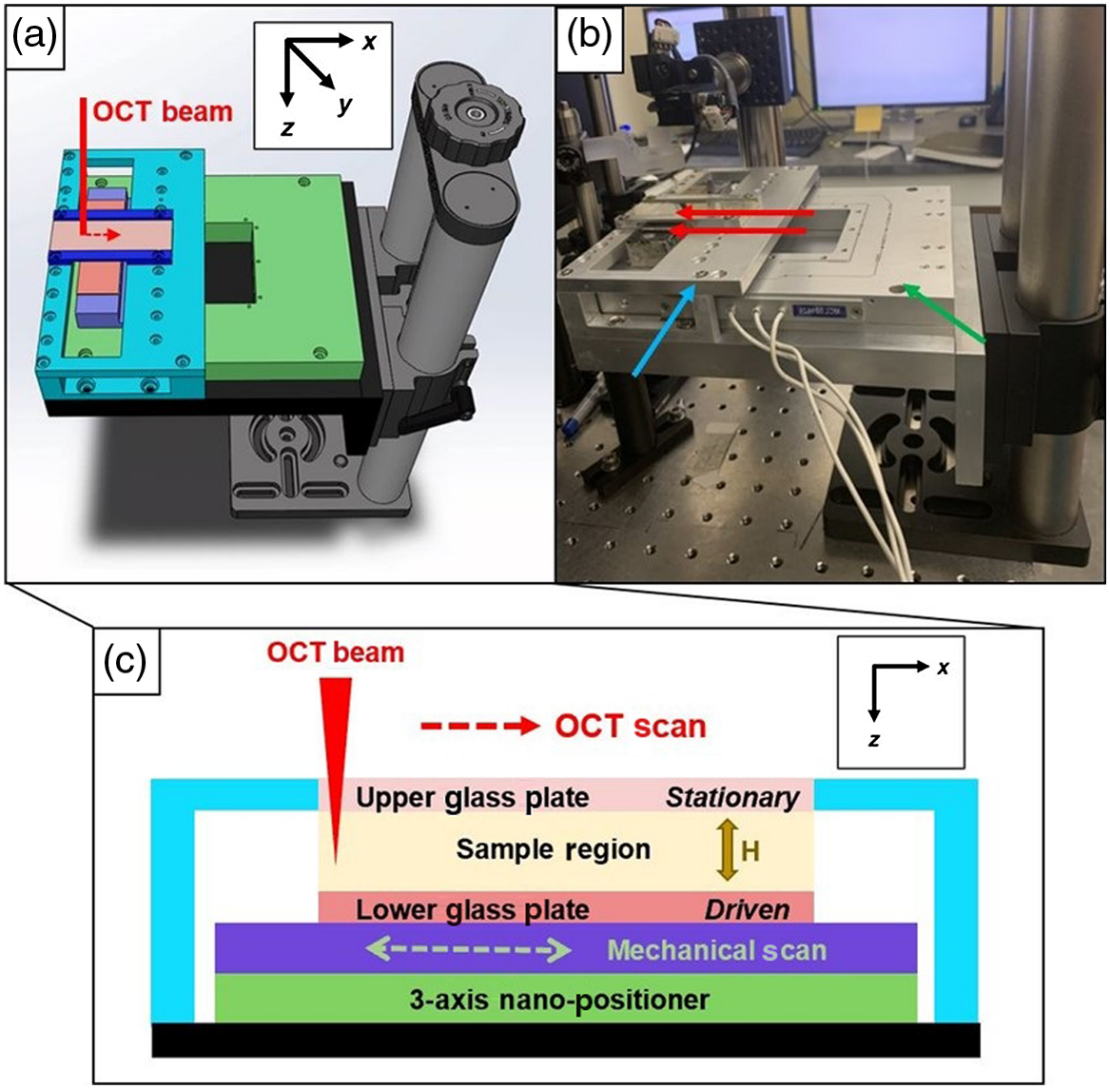
(a) Solidworks CAD design of MPPSIC on a large axial translational stage. The cage system (blue) holds the top plate stationary relative to the stage without coupling to the nanopositioner. The incoming OCT beam and scan path are indicated in red. (b) Photograph of the MPPSIC stage under the OCT sample arm. The red arrows point to the glass plates, the blue arrow points to the stationary cage system, and the green arrow points to the three-axis nanopositioner. (c) Diagram of an x–z cross section within the MPPSIC. The yellow arrow indicates plate separation distance H controlled through an external power supply. The mechanical scan and OCT scan are along the x axis as shown.

The MPPSIC consists of two removable and parallel flat plates, one of which remains stationary while the other is driven laterally with a desired waveform. To make this design amenable to OCT, the top plate is composed of optical quality 1-mm-thick glass, and the upward facing surface of the bottom plate is coated with a light-absorbing paint to avoid specular reflection. A three-axis nanopositioner (LP100 Mad City Labs, Inc.) is employed to control the bottom plate lateral oscillations and to set the distance of separation between plates. The top plate is stabilized by a cage system, uncoupled to the nanopositioner, which allows for tilt of the full strain induction chamber to reduce specular reflection from the top plate (Fig. 1).

**Fig. 2:**
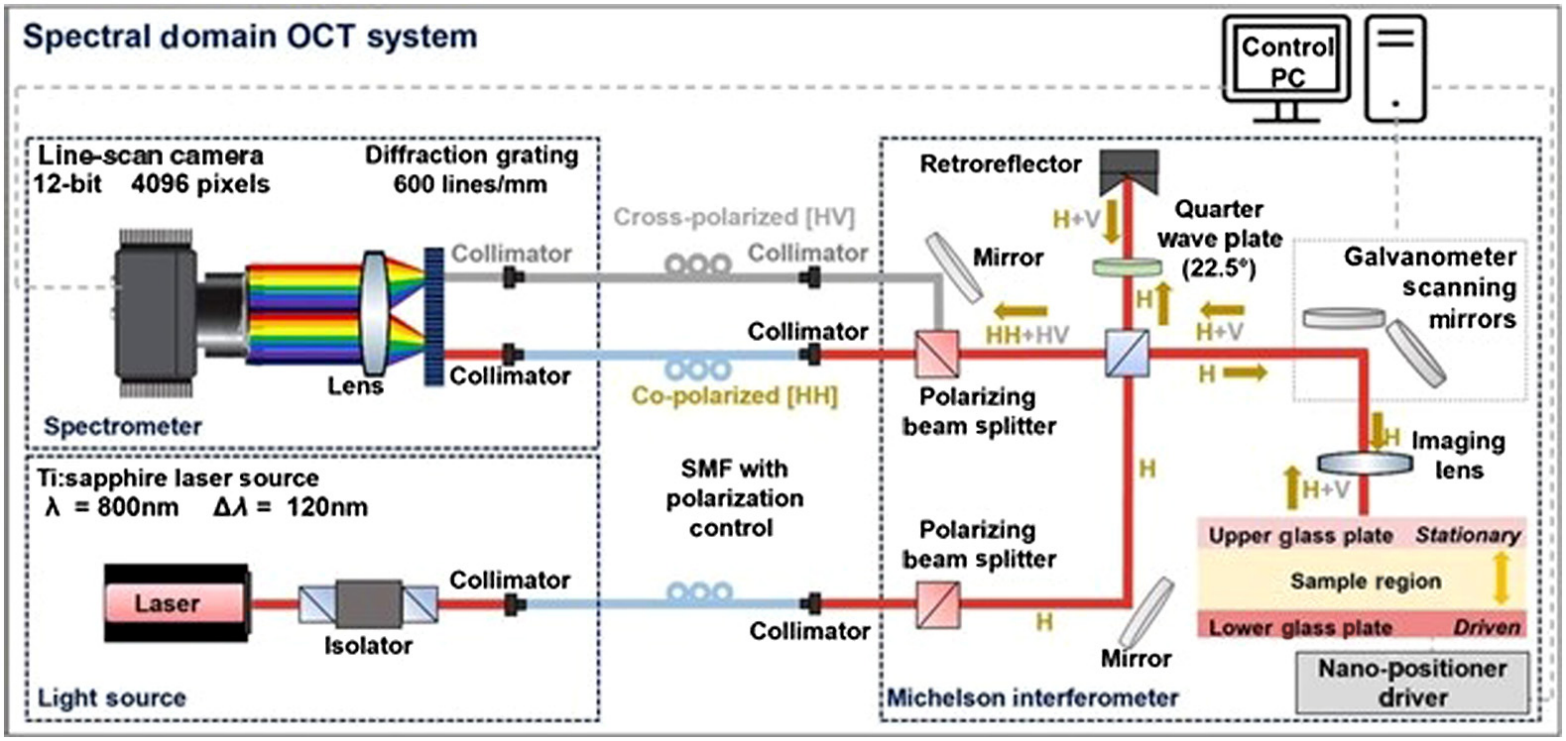
Spectral domain OCT system schematic.

The MPPSIC is designed to induce shear on liquid samples deposited between the plates. A waveform generator produces either a triangle waveform to drive the bottom plate at a constant velocity or a sinusoidal waveform to drive the bottom plate with sinusoidal velocity. The OCT data acquisition system synchronizes the start of OCT image acquisition with the start of the MPPSIC waveform, with a measured 9 ms mechanical delay accounted for image processing. The nanodriver induces lateral displacements up to 100  μm, and for waveforms driven at up to 10 Hz, this allows us to induce maximum shear rate amplitudes of 6 to $39\;s^{-1}$ on the sample while controlling the plate separations between 80 and 500  μm.

Importantly, this design can mimic the shear strain and strain rate applied to mucus by cilia at the bronchial epithelium. Mucus layer thickness is typically 20 to 100  μm, and the average length of cilia is between 9 and 12  μm with beat frequencies between 10 and 20 Hz. As such, it is estimated that mucus undergoes 1 to $10\;s^{-1}$ shear strain rates at bulk flow speeds of 60 to 100  μm/s.^25^ MCC velocities are well described by oscillations about constant velocities. Since the oscillations alone contribute to the strain within the fluid, we choose to drive our system with a sinusoidal waveform for mucus samples to mimic the shear strains and strain rates associated with MCC.^26^

### 3.2 Sample Preparation

For experiments on Newtonian fluids, three aqueous glycerol (Sigma Aldrich 99% pure glycerol) solutions were prepared (two for triangle and one for sinusoidal actuation) with 2.07  μm diameter polystyrene microspheres (Bangs Laboratories, IN) added as tracer particles. Desired volumes of water, glycerol, and microspheres solutions were calculated to target samples with a dynamic viscosity of ∼400  mPa s using equations found in Ref. 27 and a final microsphere density of 1% to 3% solids by volume. Each pipetted volume was weighed to determine the actual ratio of water to glycerol to calculate each sample’s dynamic viscosity, which were all between 294 and 462 mPa s. The refractive indices of the aqueous glycerol solutions, needed for calibration of depth in the OCT images collected at 800 nm wavelength, were calculated with the Arago-Biot formula^28^ as the summation of the weight fractions of water and glycerol multiplied by their respective refractive indices, 1.340 for water and 1.465 for glycerol.^29^ Sample refractive indices ranged between 1.45 and 1.46. For experiments in mucus, which is non-Newtonian, mucus is harvested from human bronchial epithelium cell cultures (provided by the Cystic Fibrosis/Pulmonary Research Treatment Center at the University of North Carolina at Chapel Hill) and prepared to a defined concentration following established protocols.^30^ These samples were diluted with 1× DPBS (distilled phosphate buffered saline) and microspheres were added such that the final concentration of mucus was 1.5 wt. % solids, with microsphere solids making up 1% to 3% of the sample’s total volume. Mucus samples were assumed to have a refractive index of 1.34, the same as that of water.

### 3.3 OCT System

Details of the custom polarization sensitive spectral domain OCT system used in this study were previously described.^31^ The light source of this system is an 800 nm center wavelength Ti:Sapphire laser (Griffin; KM Labs) with a 125-nm bandwidth. The power directed on the sample was ∼5  mW. The incident beam on the sample is linearly polarized light (H), and the backscattered beam components (H, V) are separated at the output of a Michelson interferometer before being imaged onto a 4096-pixel line scan camera. In the experiments described in this paper, only the co-polarized (HH) signal is used, and the cross-polarized (HV) path is blocked. The OCT system’s axial and transverse resolutions in air are 3 and 12  μm, respectively.

### 3.4 Experimental Procedures

#### 3.4.1 MPPSIC constant velocity Newtonian sample procedure

To test the functionality of the MPPSIC, we began with constant velocity experiments via triangular waveforms. Samples were loaded into the MPPSIC, and the plate separation was set to ∼300  μm. The MPPSIC was driven in the lateral direction (x) with a triangle waveform in which the speeds of the forward and backward sweeps were equal. Experiments were performed on two separate samples: sample 1 with a viscosity of 462 mPa s and sample 2 with a viscosity of 294 mPa s. Each sample was driven at 0.5 Hz with bottom plate speeds from 5 to 60  μm/s in increments of 5  μm/s. This range of experimental conditions corresponds to peak-to-peak shear strains between 1.7% and 20% and shear rates between 0.017 and $0.20\;s^{-1}$. OCT imaging was triggered at the beginning of MPPSIC oscillations, where B-mode images (1.5  mm×1.5  mm in x×z in the sample) composed of 250 A-lines were collected sequentially at a line rate of 10 kHz with 2 ms of dead time between frames, and the frame rate was 37 Hz. A total of three waveform cycles were collected in each experiment.

#### 3.4.2 MPPSIC sinusoidal velocity Newtonian sample procedure

To test the MPPSIC with a waveform that more closely recapitulates the shear dynamics of bronchial mucus, we performed experiments with the shear being sinusoidally varying. A Newtonian fluid sample of 347 mPa s was loaded into the MPPSIC, and the plate separation was set to ∼150  μm. The MPPSIC was driven in the lateral direction (x) with a sinusoidal waveform, and data were collected at driving frequencies in increments of 1 Hz from 1 to 10 Hz with a maximum bottom plate peak-to-peak displacement of 24  μm, resulting in peak speeds from 75 to 750  μm/s and shear rate amplitudes between 0.5 and $5.0\;s^{-1}$. OCT imaging was triggered at the beginning of MPPSIC oscillations, where B-mode images (1.2  mm×1.5  mm in x×z in the sample) composed of 208 A-lines were collected sequentially at a line rate of 69 kHz; with 2 ms of dead time between frames, and the frame rate was 199 Hz. A total of six waveform cycles were collected in each experiment.

#### 3.4.3 MPPSIC sinusoidal velocity mucus sample procedure

To observe motion tracking capabilities in mucus, experiments were performed on a bronchial mucus sample. Mucus prepared as above was loaded into the MPPSIC, and the plate separation was set to ∼240  μm. The MPPSIC was driven in the lateral direction (x) with a sinusoidal waveform. Eight experiments were performed on the sample using driving frequencies in increments of 1 Hz from 3 to 10 Hz. Each sample was driven with a maximum bottom plate peak-to-peak displacement of 18  μm and then repeated at 24  μm, resulting in peak speeds from 170 to 750  μm/s and shear rate amplitudes between 0.7 and $3.2\;s^{-1}$. OCT imaging was triggered at the beginning of MPPSIC oscillations, where B-mode images (1.2  mm×1.5  mm in x×z) composed of 208 A-lines were collected sequentially at a line rate of 69 kHz; with 2 ms of dead time between frames, and the frame rate was 199 Hz. A total of six waveform cycles were collected in each experiment.

## 4 Image Analysis

**Fig. 3:**
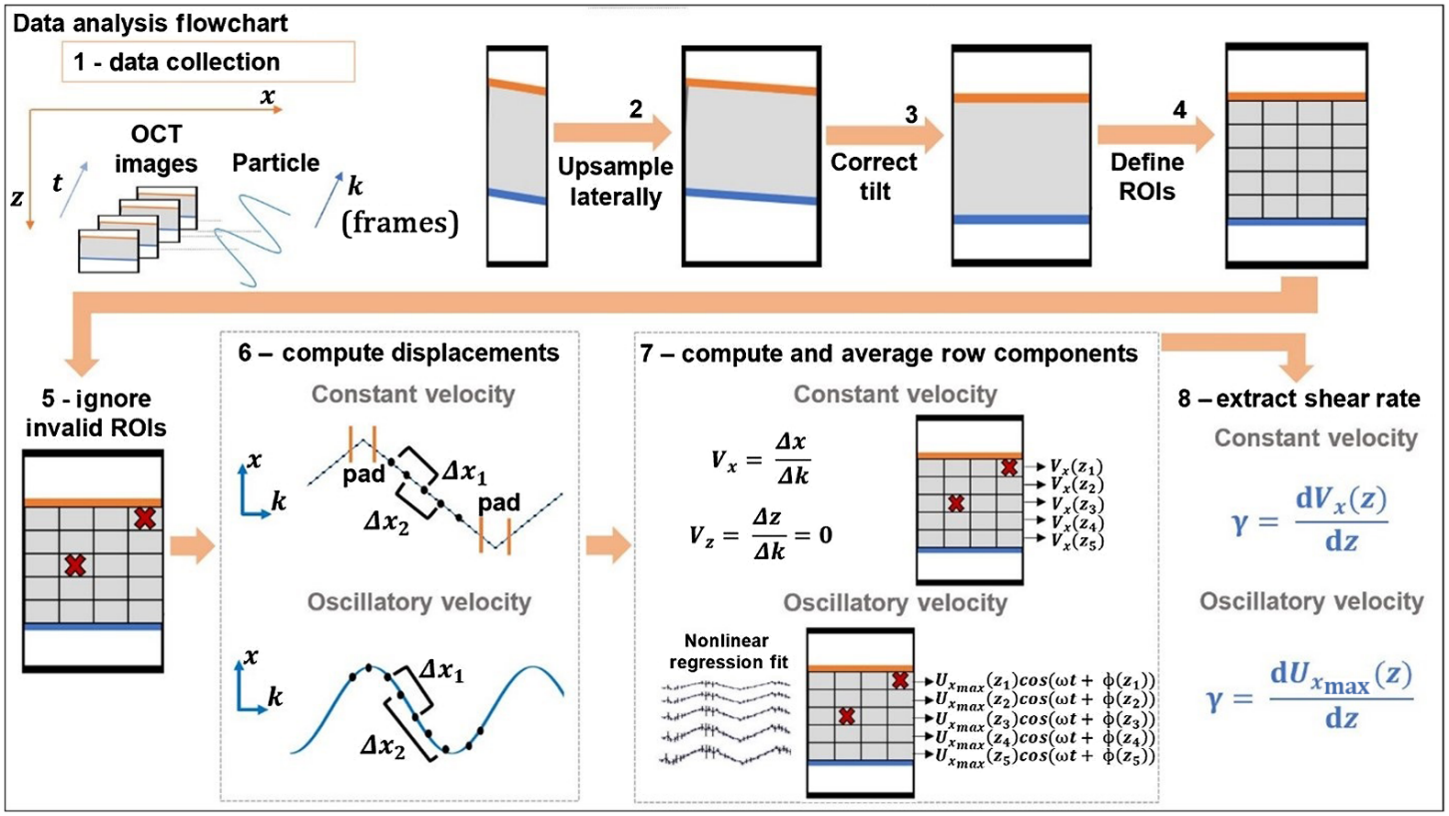
Image analysis flowchart. Step 1 is performed with LabView, and all other steps were performed in MATLAB 2019b. To omit ROIs that contain no particles to track, we employ two steps: intensity thresholding and then a cluster check. A threshold is calculated for each row i of ROIs to account for system sensitivity roll-off. The threshold is the mean pixel intensity (averaged over j, k) plus 1.5× the standard deviation. Thresholded ROIs that contain more than one non-zero pixel are then tested to determine if the intensity is spatially clustered, suggesting the presence of a particle. This is performed by autocorrelating each thresholded ROI with a 1-pixel diagonal displacement and keeping only ROIs for which the result is non-zero. Each ${\mathrm{ROI}}_{i,j}$ must pass the cluster test across all frames (k) to be considered non-empty. Also each row of ROIs must contain at least one non-empty ROI for the image stack to be considered valid for analysis.

After collecting OCT images of fluids within the MPPSIC, the goal is to accurately extract the microsphere displacements (and corresponding velocities) in depth and time, for subsequent comparison against existing models of fluid dynamics. A flowchart of the image analysis procedure to extract $V_{x}(z,t)$ using a normalized cross-correlation method is shown in Fig. 3.

### 4.1 Region of Interest Selection

The frames corresponding to the first 30 ms are discarded to avoid tracking transient motions induced by the system as well as the MPPSIC’s mechanical lag. To mitigate digitization noise, all images are upsampled in the lateral direction (x) by a factor of 4. Then the tilt of the plates from horizontal, which is necessary in OCT to avoid strong specular reflections that saturate the detection hardware, is corrected by a semiautomated method. The top plate’s angle is used to vertically shift each column of the image to form a horizontal surface, and the difference between the top and bottom plates’ angles is checked to ensure that the plates are parallel within 1 deg. Throughout the image analysis procedure, OCT pixel sizes calibrated in free-space are multiplied by an axial distortion factor $D_{\text{axial}}=\frac{\sqrt{\left(n^{2}-\sin^{2}(\alpha )\right)}}{n^{2}}$ and transverse distortion factor $D_{\text{transverse}}=\frac{1}{\cos(\alpha )}$ to account for the effects of the sample refractive index n determined as above and tilt angle α. After this correction, the separation distance H between the top and bottom surfaces is measured at the center of the image.

The sample region is divided into a grid of ROIs of i=1. N rows by j=1. M columns for each frame k=1…Q denoted as $\mathrm{ROI}{\left(k\right)}_{i,j}$. ROI dimensions were chosen to provide sufficient resolution in the axial direction (15 to 18  μm) for shear analysis, while extending further in the lateral (x) direction (150  μm) to capture ∼1 to 4 microspheres per ROI to facilitate cross-correlation measurements. ROIs were 12×100 or 18×100  pixels for experiments with large (>200  μm) or small (<200  μm) plate separations, respectively.

Displacements are obtained within each non-empty ROI by normalized cross correlation. Correlation coefficients are computed between pairs of thresholded ROIs within the time series, $I_{1}(x,z)=\mathrm{ROI}{\left(k_{1}\right)}_{i,j}$ and $I_{2}(x,z)=\mathrm{ROI}{\left(k_{2}\right)}_{i,j}$ according to

$$\rho (u,v)=\frac{\underset{x,z}{\sum }[I_{1}(x,z)-\overset{¯}{I_{1}}][I_{2}(x-u,z-v)-\overset{¯}{I_{2}}]}{{\left\{\underset{x,z}{\sum }{\left[I_{1}(x,z)-\overset{¯}{I_{1}}\right]}^{2}\underset{x,z}{\sum }{\left[I_{2}\left(x,z\right)-\overset{¯}{I_{2}}\right]}^{2}\right\}}^{\left(\frac{1}{2}\right)}},$$ (5)

where $I_{2}$ [and hence the range of (u,v) computed] is extended by the theoretical lateral (in x) and axial maximum displacement of a particle. The (u,v) corresponding to the maximum correlation ρ is taken as the lateral and axial particle displacement $\left(x_{\text{shift}},z_{\text{shift}}\right)$, respectively. The $z_{\text{shift}}$ values were found to be consistently close to zero pixels and were considered negligible.

### 4.2 Constant Velocity Image Analysis

For constant velocity experiments, a triangle waveform drives the bottom plate. To avoid transient effects in the stage or fluid at the turnaround points of the waveform, we omit (“pad”) all OCT images within two frames of the waveform peaks and valleys.

As depicted in Fig. 3, each half cycle of the waveform is analyzed by computing displacements between pairs of frames. Pairs were chosen by employing a depth-dependent frame decimation value $\Delta k_{i}$ based upon an initial estimate of the velocity within that row. Details of the decimation technique may be found in the Supplementary Material. The purpose of the decimation is to exaggerate motion in regions of low velocity, such as near the top plate, where displacements between successive frames can be less than a single pixel. Initial velocities are estimated by linear extrapolation of the velocity obtained at the bottom in ${\mathrm{ROI}}_{N,j}$ to the top plate where we expect a velocity of zero.

Finally, lateral displacements $x_{\text{shift}}{\left(k\right)}_{i,j}$ are computed within each sweep by cross correlating $I_{1}=\mathrm{ROI}(k{)}_{i,j}$ with $I_{2}=\mathrm{ROI}(k+\Delta k_{i}{)}_{i,j}$ for $k=1\ldots F-\Delta k_{i}$ according to Eq. (5), where F is the number of frames in one sweep. We then compute the velocity $V_{x}$ in pixels per frame according to

$$V_{x}{\left(k\right)}_{i,j}=\frac{x_{\text{shift}}{\left(k\right)}_{i,j}}{\Delta k_{i}}.$$ (6)

In each ${\mathrm{ROI}}_{i,j}$, the velocity in each half cycle q is computed by averaging $V_{x}{\left(k\right)}_{i,j}$ over all k resulting in $V_{x}{\left(q\right)}_{i,j}$. The average and standard deviation of the speed in each row i are then computed from the absolute value (to account for forward and backward motions) of $V_{x}{\left(q\right)}_{i,j}$ over all j and q, providing $V_{x}(z)=V_{xi}\pm \sigma_{Vxi}$.

### 4.3 Sinusoidal Velocity Image Analysis

For sinusoidally varying velocity experiments, the driving velocity is no longer constant, so the approach above is extended to account for variable frame decimation in time $\Delta k{\left(k\right)}_{i}$. The details of the decimation technique may be found in the Supplementary Material. The frame decimation value for each frame and depth $\Delta k{\left(k\right)}_{i}$ is then used to define each pair of images $I_{1}=\mathrm{ROI}{\left(k\right)}_{i,j}$ and $I_{2}=\mathrm{ROI}{\left(k+\Delta k{\left(k\right)}_{i}\right)}_{i,j}$, where k is now extended over all frames in the scan (unlike triangle data analyzed within each half sweep). Analogous to Eq. (6), velocities $U_{x}{\left(k+\frac{\Delta k(k{)}_{i}}{2}\right)}_{i,j}$ are computed by dividing $x_{\text{shift}}(k{)}_{i,j}$ by $\Delta k{\left(k\right)}_{i}$. Note that, since the velocity is now varying in time, each velocity is assigned to a frame in the middle of the interval, located at $k+\frac{\Delta k{\left(k\right)}_{i}}{2}$. Finally, the velocity in each row of ROIs is averaged, while preserving the time dependence (k), to obtain an average velocity waveform at each depth:

$$U_{x}{\left(k\right)}_{i}=\frac{\sum_{j=1}^{M}U_{x}{\left(k\right)}_{i,j}}{M},$$ (7)

and velocities are converted from units of pixels per frame to microns per second according to the distortion factors above.

To extract velocity amplitude, frequency, and phase, velocity waveforms were fit by non-linear least squares to a sinusoidal waveform. For rows with only one valid ROI, an unweighted fit was used; for rows with multiple valid ROIs, a weighted fit was used, with the standard deviations of the $U_{x}{\left(k\right)}_{i,j}$ used as the weights.

## 5 Experimental Results

### 5.1 MPPSIC Constant Velocity Performance on Newtonian Fluid

**Fig. 4:**
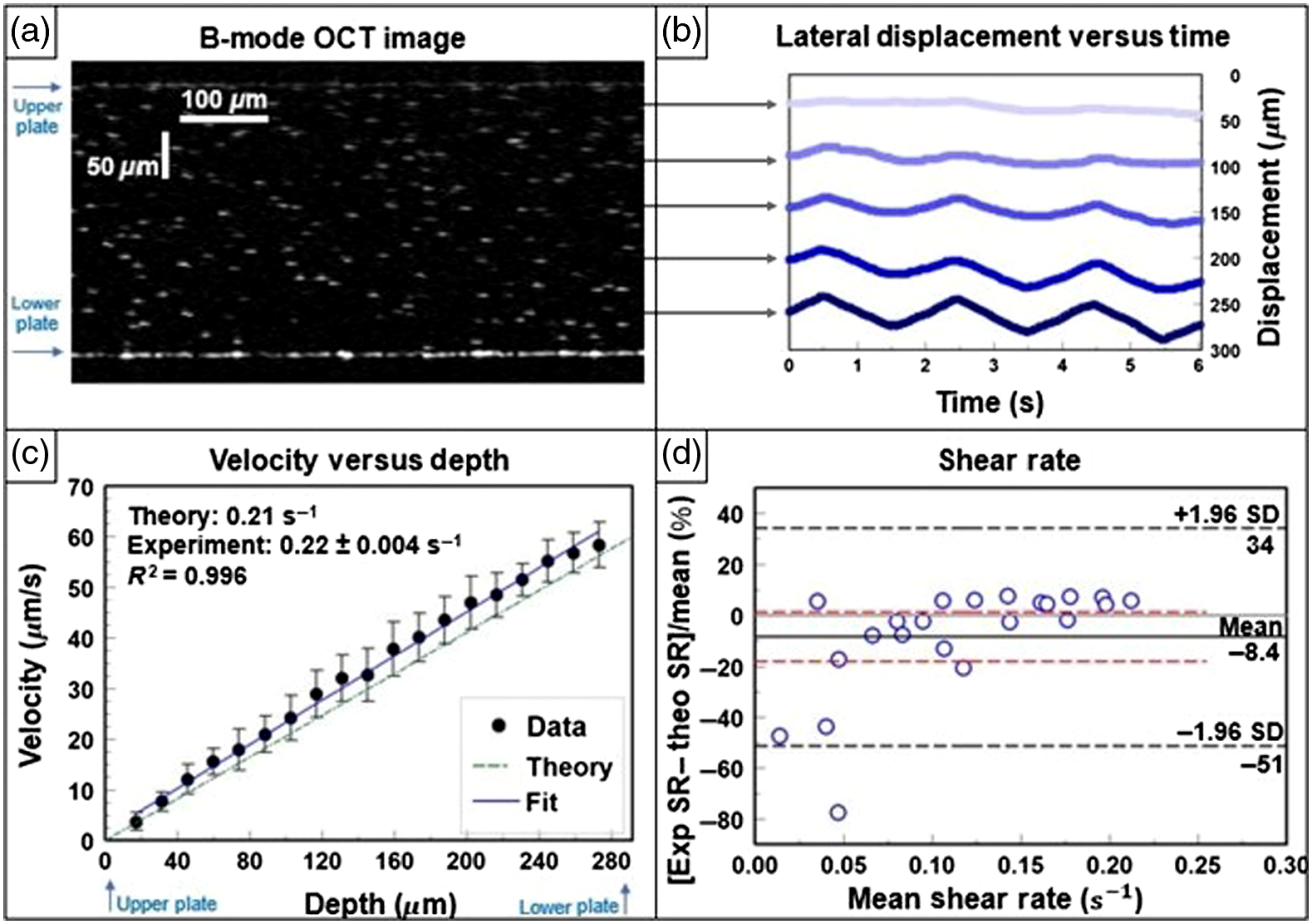
Newtonian fluid under constant shear rate in the MPPSIC. (a) OCT image of sample 2, a glycerol:water solution of 294 mPa s viscosity containing polystyrene microspheres as tracer particles between the glass plates of the MPPSIC separated by 292  μm. Image is cropped to 0.65 mm in the lateral direction (x) for clarity. (b) Corresponding traces of lateral particle displacements in sample 2 when driven by a triangle waveform at the bottom plate with peak velocity of 60  μm/s and frequency of 0.5 Hz. Traces are shown at multiple depths in the sample, offset by their depth position for clarity. Corresponding movie of B-mode OCT images (1.4  mm×0.4  mm in x×z) displayed at 2× real time (Video 1, MP4, 2.9 MB [URL: https://doi.org/10.1117/1.JBO.26.9.096005.1]). (c) Corresponding waveform-averaged tracked particle velocity versus depth; the best-fit line is used to extract the shear rate, which is compared with theory. (d) Bland–Altmann plot comparing results of measured shear rates over all experiments (n=22) compared with theoretical shear rates. The difference is expressed as a percentage on the y axis. The LoAs are marked by black dashed lines, the bias by a solid line, and 95% confidence interval of the bias by red lines. Velocity was varied while all other parameters were held constant for each sample’s data collection.

In Fig. 4(a), an example OCT B-mode image frame shows the distribution of the microsphere tracer particles within a glycerol–water sample between the glass plates. To showcase the motion tracking capabilities of our method, in Fig. 4(b), the average particle displacement versus time is plotted for a handful of depths for one of the experiments. The pattern of the particle displacement clearly follows the triangle waveform driving the bottom plate, and the amplitude of displacement gradually increases proportionally to distance from the stationary plate, which is consistent with theory. To extract a shear rate, the particle velocity versus depth [Fig. 4(c)] is plotted for this same experiment. The theoretical trend line for the shear rate is displayed, and a weighted linear regression is set to the data. The slope of the weighted fit is extracted as the shear rate [Eq. (2)] to be $0.22\pm 0.004\;s^{-1}$, compared with the theoretical shear rate of $0.21\;s^{-1}$, and the r-squared value of the weighted least squares regression is 0.996. In Fig. 4(d), the results of all 22 experimental shear rates, obtained by varying the velocity while keeping all other parameters constant, are compared with the theoretical shear rates via a Bland–Altmann plot. Difference values are expressed as percentages to better represent the relationship between experimental and theoretical results. Results of the Bland–Altmann plot are summarized in Table 1. The bias is −8.4% (95% CI, −18% to 1.2%) within the limits of agreement (LoA) range of −51% to 34% that is principally caused by the lower shear rate measurements. Above $0.05\;s^{-1}$, the agreement range is <20%.

**Table 1:** Results of constant velocity and sinusoidal velocity experiments.

		Bias (%)	Lower confidence interval (%)	Upper confidence interval (%)	Lower limit of agreement (%)	Upper limit of agreement (%)
Constant velocity	Aqueous glycerol sample	−8.4	−18	1.2	−51	34
Sinusoidal velocity		1.6	−0.28	3.4	−3.5	6.7
	Mucus sample	−4.9	−7.2	−2.6	−13	3.6

### 5.2 MPPSIC Sinusoidal Velocity Performance on Newtonian Fluid

**Fig. 5:**
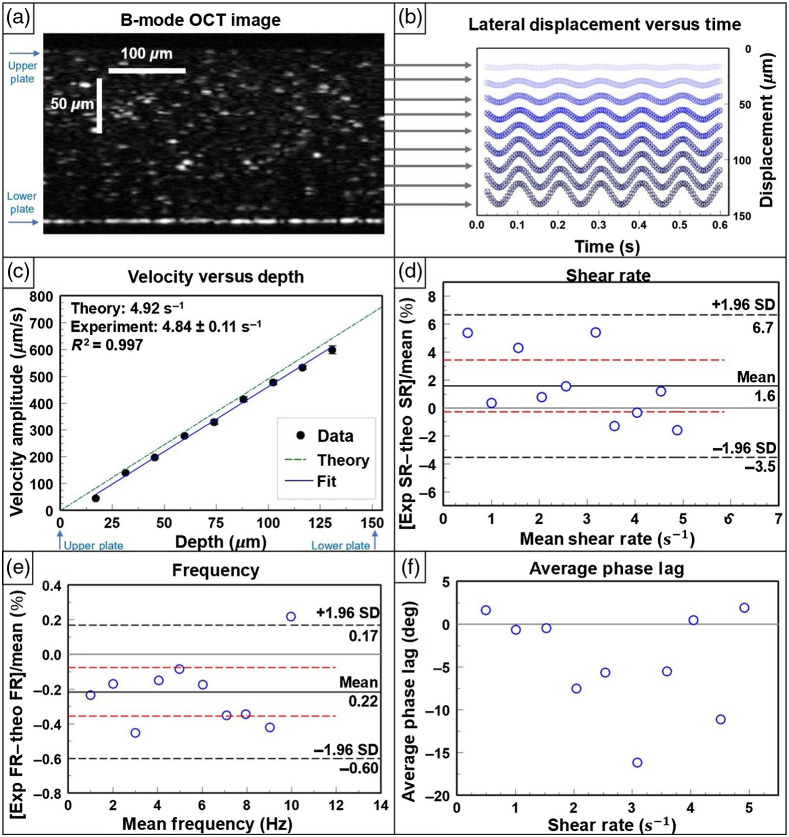
Newtonian fluid under sinusoidal shear in the MPPSIC. (a) OCT image of a glycerol:water solution of 347 mPa s viscosity containing tracer particles in the MPPSIC with a glass plate separation of 154  μm. Image is cropped to 0.45 mm in the lateral direction (x) for clarity. (b) Corresponding traces of lateral particle displacements when driven by a sinusoidal waveform at the bottom plate with peak-to-peak displacement of 24  μm and frequency of 10 Hz. Traces are shown at multiple depths in the sample, offset by their depth position for clarity (5 of 6 waveforms shown). Corresponding movie of B-Mode OCT images (1.06  mm×0.23  mm in x×z) displayed at 0.33× real time (Video 2, MP4, 1 MB [URL: https://doi.org/10.1117/1.JBO.26.9.096005.2]). Error bars are not visible due to small size. (c) Example waveform-averaged tracked particle velocity amplitude versus depth; the best-fit line is used to extract the shear rate amplitude, which is compared with theory. Error bars are not visible due to small size. (d) Bland–Altmann plot comparing results of measured shear rate amplitudes over all experiments (n=10) compared with theoretical shear rate amplitude. The difference is expressed as a percentage on the y axis. The LoAs are marked by black dashed lines, the bias by a solid line, and 95% confidence interval of the bias by red lines. Frequency was varied while all other parameters were held constant for each sample’s data collection. (e) Bland–Altmann plot comparing results of measured frequency over all experiments (n=10) compared with theoretical frequency. The difference is expressed as a percentage on the y axis. (f) Corresponding results of measured weighted average phase lag compared with theory (φ=0).

In Fig. 5(a), an example OCT B-mode image frame again shows the distribution of the microsphere tracer particles between the plates, now with a smaller separation distance. Taking a representative experiment, we see the sinusoidal pattern of the average particle displacement versus time in Fig. 5(b) and note that the amplitude of displacement increases proportionally to distance from the stationary plate. In Fig. 5(c), the fitted velocity amplitude for this experiment is plotted versus depth. The theoretical trend line for the shear rate amplitude is displayed, and a weighted linear regression is set to the data. The slope of the weighted fit is extracted as the shear rate amplitude [Eq. (4)] to be $4.84\pm 0.11\;s^{-1}$, compared with the theoretical shear rate amplitude of $4.92\;s^{-1}$, and the weighted fit has an r-squared of 0.997. In Fig. 5(d), the results of all 10 experimental shear rate amplitudes, obtained by varying the frequency while keeping all other parameters constant, are compared with the theoretical shear rate amplitudes (bias=1.6%; 95% CI, −0.28% to 3.4%; LoA, −3.5% to 6.7%). Similarly, the experimentally extracted frequencies are compared with corresponding theoretical values for all 10 experiments [Fig. 5(e)], and the phase lag of each experiment, calculated by a weighted average, is shown in Fig. 5(f).

### 5.3 MPPSIC Sinusoidal Velocity Performance on Mucus

**Fig. 6:**
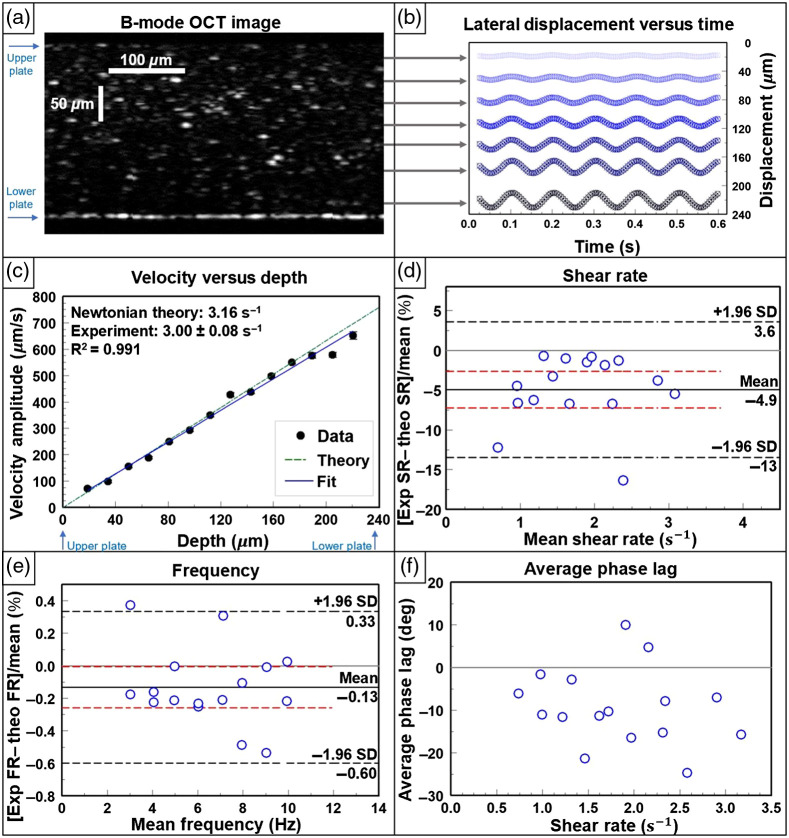
1.5 wt. % mucus under sinusoidal shear in the MPPSIC. (a) OCT image of a mucus sample containing polystyrene tracer particles in the MPPSIC with a glass plate separation of 242  μm. Image is cropped to 0.45 mm in the lateral direction (x) for clarity. (b) Corresponding traces of lateral particle displacements when driven by a sinusoidal waveform at the bottom plate with peak-to-peak displacement of 24  μm and frequency of 10 Hz. Traces are shown at multiple depths in the sample, offset by their depth position for clarity (5 of 6 waveforms shown). Corresponding movie of B-mode OCT images (1.06  mm×0.32  mm in x×z) displayed at 0.33× real time (Video 3, MP4, 1 MB [URL: https://doi.org/10.1117/1.JBO.26.9.096005.3]). (c) Example waveform-averaged tracked particle velocity amplitude versus depth; the best-fit line is used to extract the shear rate amplitude, which is compared with Newtonian theory (noting that mucus is, in general, non-Newtonian). Error bars are not visible due to small size. (d) Bland–Altmann plot comparing results of measured shear rate amplitudes over all experiments (n=16) compared with Newtonian theoretical shear rate amplitudes. The difference is expressed as a percentage on the y axis. The LoAs are marked by black dashed lines), the bias by a solid line, and 95% confidence interval of the bias by red lines. (e) Bland–Altmann plot comparing results of measured frequency over all experiments (n=16) compared with theoretical frequency. The difference is expressed as a percentage on the y axis. (f) Corresponding results of measured weighted average phase lag compared with theory (φ=0).

In Fig. 6(a), an example OCT B-mode image frame shows the dispersion of the polystyrene microspheres within the 1.5 wt. % mucus sample, where additional scatterers endogenous to the mucus are also evident in the image. For one experiment, we see the sinusoidal pattern of the average particle displacement versus time in Fig. 6(b); similar to the results for the glycerol–water solutions, we find that the amplitude of displacement increases proportionally to distance from the stationary plate. In Fig. 6(c), the fitted velocity amplitude for this experiment is plotted versus depth. To assess if there is a difference from Newtonian behavior, the Newtonian theoretical trend line for the shear rate amplitude is displayed, and a weighted linear regression is set to the data. The slope of the weighted fit is extracted as the shear rate amplitude [Eq. (4)] to be $3.00\pm 0.08\;s^{-1}$, compared with a theoretical shear rate amplitude (if Newtonian) of $3.16\;s^{-1}$, and the weighted fit has an r-squared of 0.991. Finally, the experimentally extracted shear rate amplitudes are compared with (Newtonian) theoretical shear rate amplitudes (bias=−4.9%; 95% CI, −7.2% to −2.6%; LoA, −13% to 3.6%), and the experimental derived frequencies are compared with corresponding theoretical values for all 16 experiments [Figs. 6(d) and 6(e)]. The phase lag of each experiment was also calculated and shown in Fig. 6(f). The phase lag of each row was minimal (<20  deg) and considered negligible.

## 6 Discussion

In this study, two sets of experiments were conducted with Newtonian fluids to test the functionality of the MPPSIC and develop motion tracking methods. A third set of experiments was conducted to observe the motion tracking capabilities of our method in a biofluid consisting of 1.5 wt. % bronchial mucus. As shown in Fig. 4, our method tracks particle displacement, allowing us to extract the velocity of particles at various known depths when under constant velocity conditions. The depth-resolved velocities are consistent with a linear trend line [Fig. 4(c), $R^{2}=0.996$] as expected for a homogeneous, Newtonian fluid at a low Reynolds number. The slope of the linear fitting for each depth-resolved experiment was used to experimentally determine the shear rate, which showed good correspondence [Fig. 4(d)] with the theoretical shear rate. However, the large LoAs range in Fig. 4 may be explained by the larger error that our algorithm had in tracking shear rates lower than $0.05\;s^{-1}$. Error in trials considered “valid” by the algorithm may be the result of autocorrelation artifacts in the OCT image that appear near the top plate and result in false tracking or a lack of significant motion with lower waveform velocities. In the case of a slow-moving bottom plate, even with up-sampling and frame decimation techniques, there may not have been enough frame-to-frame motion for adequate tracking. Trials in the experiment were unreported if considered invalid by the algorithm, such as when the analysis method was unable to proceed due to either the presence of large autocorrelation artifacts, which fails to pass the cluster check, or when the microspheres migrated toward the top plate, which fails to have a valid ROI in each depth.

As shown in Fig. 5, the motion tracking algorithm performs well at quantifying shear rate amplitudes under sinusoidal shear in conditions close to those found in MCC (∼1 to $10\;s^{-1}$), which are obtained when using a small plate separation and high frequencies. The tracked motion of particles within the fluid follows an expected sinusoidal displacement in time and constant gradient in depth as expected for a homogeneous Newtonian fluid at a low Reynolds number. Our analysis method extracts velocity amplitude, frequency, and phase by fitting the average velocity waveform at each ROI depth. Figure 5(c) provides an example of results of one experiment, in which we can see that the experimentally captured shear rate amplitude of $4.84\pm 0.11\;s^{-1}$ is consistent with the theoretical shear rate amplitude of $4.92\;s^{-1}$. In Fig. 5(d), we show that the MPPSIC algorithm accurately extracts shear rate amplitudes at up to $5\;s^{-1}$, which is within the range typically induced by cilia in the MCC. The line of equality falls within the 95% confidence interval of the bias, and the LoAs are a narrow range of <10%. However, the same potential for analysis errors exists in the oscillating velocity experiment as under constant velocity conditions. In addition to the abovementioned contributions to error, we also become limited by the available frame rate of our OCT system, which is ∼200  Hz when capturing ∼200 A-lines as in these experiments. This contributed to error in the higher frequency experiments (∼8  Hz and above), as the number of frames per waveform cycle became too low for adequate particle tracking. A potential future solution to this frame rate limitation would be to employ line field OCT to image at kilohertz frame rates,^32^^–^^34^ with the added benefit of avoiding lateral beam scanning, which can confound the analysis of image frames of moving particles.

Mucus samples were used for initial observations of MPPSIC particle tracking within a biofluid. Mucus concentrations are considered to be in a normal hydrated state at concentrations ∼1.5 to 3 wt. %. At a 1.5 wt. % concentration, our mucus can be considered a sample representing the mucus in a well hydrated state.^15^ Similar to the Newtonian fluid sinusoidal experiments, these experiments were conducted using shear rate amplitudes (up to $3\;s^{-1}$) extending into the range expected in the respiratory epithelium. However, the plate separation was somewhat higher (∼240  μm) than the typical height of the mucus layer in the respiratory epithelium (∼20 to 100  μm). The experiment shown in Figs. 6(a)–6(c) was selected for display as it was performed at the highest velocity amplitude and thus was most challenging to the motion-tracking algorithm. It was found that the time course of the mucus motion appears to be sinusoidal at the fundamental frequency of actuation as well as in-phase with the actuation, suggestive of a linear elastic response of the mucus. A non-zero phase lag, particularly near the central depths of the sample, would be expected in the case of a non-linear depth profile; however, the phase lag was negligible across all experiments. Furthermore, the mucus follows a linear velocity amplitude profile in depth [$R^{2}=0.991$ for the experiment shown in Fig. 6(b)], similar to Newtonian samples. Using the sinusoidal analysis method, we again extracted velocity amplitude, frequency, and phase from the average velocity waveform at each ROI depth. The example experimental data in Fig. 6(c) indicate that the experimentally captured shear rate amplitude of $3.00\pm 0.08\;s^{-1}$ is similar to, but not entirely consistent with, the Newtonian theoretical shear rate amplitude of $3.16\;s^{-1}$. Altogether, the data across all shear rate amplitudes [Fig. 6(d)] are consistently underestimated in comparison with Newtonian theory, with the line of equality falling outside the bias 95% confidence interval (−7.2% to −2.6%).

Our observations of the mucus velocity waveforms suggest that the shear strain amplitude applied to the mucus was not large enough to induce a non-linear elastic response, such as shear-thinning that might be expected for mucus. At a peak-to-peak displacement amplitude of 24  μm and plate separation of ∼240  μm, there is ∼10% shear strain on the sample, which is on the boundary between small amplitude oscillation shears and large amplitude oscillation shears,^26^ which may be required to measure non-linear responses in mucus. In addition, our observations of linear velocity amplitude profiles of mucus in depth are consistent with that expected for non-Newtonian fluids within the gap-loading limit, i.e., when the plate separation is too small to support bidirectional shear waves. The maximum plate separation that lies within the gap-loading limit increases with the bulk shear modulus of the fluid and decreases with the frequency of actuation. Thus even though mucus is generally a non-Newtonian fluid, the velocity amplitude depth profiles would be identical to that of a Newtonian fluid if experiments were conducted within the gap-loading limit.^26^

These first observations in biofluids indicate that we can track the depth dependence of flow in mucus samples under conditions that mimic the frequency and shear amplitude of the airway epithelium. With the capability of tuning each of these parameters individually, the MPPSIC is ideal for the exploration of fluid flow in physiologic conditions. Here we have shown our algorithm tracks mucus flow across a relatively large dynamic range of flow velocities (5 to 700  μm/s) and over hundreds of microns in depth. In future experiments, we will employ plate separations as low as 80  μm and deformation amplitudes as high as 100  μm, potentially quantifying how the viscoelastic and shear-thinning properties of mucus alter flow patterns in regimes that have not yet been observed. Ultimately, this can provide a new platform to investigate how changes in mucus concentration, layer height, and ciliary activity in the respiratory epithelium lead to changes in fluid flow and the loss of MCC.

Another potential future application of the MPPSIC is performing quantitative rheology on biofluids, which may be possible via the methods described in Mitran et al.^35^ In the case of bronchial mucus as studied here, quantitative rheology may be feasible using larger plate separations and/or larger frequencies than those used in this study to move outside the gap-loading limit. The ability to use OCT with the MPPSIC to actuate and depth-resolve biofluid flow, and subsequently to analyze the flow patterns with an extension of the Ferry shear wave model at a finite depth,^35^ presents a new opportunity to quantify the viscoelastic properties of fluids.

## 7 Conclusion

In summary, we propose an MPPSIC for conducting particle tracking experiments in Newtonian and non-Newtonian fluids under dynamic shear while imaging with OCT. Our sinusoidal shear experiments in Newtonian fluid provide a depth-dependent velocity amplitude profile that is consistent with theory, demonstrating the functionality of the MPPSIC and motion tracking method. Furthermore, our results in mucus indicate that the MPPSIC provides a new capability for studying biofluids, such as mucus, to assess shear-dependent properties in a regime that is relevant to the mucus layer in lung epithelium.

## Code, Data, and Materials Availability

Code and data can be made available upon request to the corresponding author.
