# Supplementary material for: OCT particle tracking velocimetry of biofluids in a microparallel plate strain induction chamber
Source: J Biomed Opt. 2021 Sep 15;26(9):096005. doi: 10.1117/1.JBO.26.9.096005 (PMC8441543; doi:10.1117/1.JBO.26.9.096005)
Supplement: Supplementary file 1 [file JBO_026_096005_SD001.pdf]

Supplementary Material

OCT particle tracking velocimetry of biofluids in a micro-parallel plate strain induction chamber

Kelsey J. Oeler[a], David B. Hill[b], and Amy L. Oldenburg[a,c,*]

[a]Department of Biomedical Engineering, University of North Carolina at Chapel Hill, Chapel Hill, NC 27599, USA

[b]Cystic Fibrosis/Pulmonary Research and Treatment Center, University of North Carolina at Chapel Hill, Chapel Hill, NC 27599, USA

[c]Department of Physics and Astronomy, University of North Carolina at Chapel Hill, Chapel Hill, NC 27599, USA

In this supplemental document, we provide a more detailed explanation of the experimental procedure, the tilt adjustment method, and decimation techniques used in the image analysis algorithm described in Section 4 of the main text.

1 Experimental Procedure

Table S1 provides a detailed list of key imaging parameters for the constant velocity and sinusoidal velocity experiments over all frequencies.

Table S1: Key imaging parameters for all experiments. Sinusoidal experiments were repeated with the same parameters for peak-to-peak bottom plate displacements of 18 μm and 24 μm.

**Constant Velocity Experiment**		**Sinusoidal Velocity Experiments**		
Frame Rate [Hz]	37.04	Frame Rate [Hz]	199	
Line Rate [kHz]	10	Line Rate [kHz]	69.252	
A-Lines	250	A-Lines	208	
dx scan [mm]	1.5	dx scan [mm]	1.2	
Dead Time [ms]	2	Dead Time [ms]	2	
Driving Frequency [Hz]	0.5	Peak-to-Peak Bottom Plate Displacement [μm]	18; 24	
Aqueous Glycerol Sample				Mucus Sample
Bottom Plate Speed [μm/s]	Number of B-Mode Images	Driving Frequency [Hz]	Number of B-Mode Images	Number of B-Mode Images
5	800	0	-	-
10	800	0.5	-	-
15	600	1	-	-
20	600	2	-	-
25	400	3	396	396
30	300	4	294	294
35	300	5	240	240
40	200	6	198	198
45	200	7	168	168
50	200	8	150	150
55	200	9	132	132
60	200	10	120	120

2 Image Analysis

2.1 Tilt Adjustment Method

For the tilt adjustment method, the user initially marks approximate locations of the top and bottom surfaces of the fluid. For each surface, a line is extrapolated through the user-defined points, then a 10-pixel vertical window searched to locate pixels in each column with maximum intensity. Then, new lines are fit through the intensity maxima, which are used to define the top and bottom of the sample region as well as the angles of the top and bottom plates relative to horizontal. The separation distance H between the top and bottom surfaces is measured at the center of the image. Distortion, D_{axial} and $D_{\text{transverse}}$, is accounted for in the height calculation. The surfaces are only segmented for one frame in each stack as there is negligible movement of the plates between frames.

2.2 Decimation Technique

Decimation is used to exaggerate motion in regions of low velocity, such as near the top plate, where displacements between successive frames can be less than a single pixel.

2.2.1 Constant Velocity

Frames corresponding to turn around points in velocity (velocity extrema) are identified by an initial sweep of x_{shift} (using Eqn. (5)) over successive frames of an ROI in the bottom row ($\text{ROI}_{N,j}$) and by subsequently plotting the cumulative displacement. The chosen number of 2 frames padding the extrema was based on the mechanical turn-around time of the nano-positioner.

Because we expect the velocity to decrease linearly between the bottom and top plates, we determine a row (i) dependent decimation value, Δk_i, based upon an initial estimate of the velocity within that row, v_i (in pixels / frame). Initial velocity is computed by linear regression of the depth and velocity of the bottom $\text{ROI}_{N,j}$ and the top of the sample region, where we expect a velocity of zero. To target a displacement of at least 3 pixels, we define Δk_i as $\frac{3}{V_i}$ or 1, whichever is larger. In high velocity regions Δk_i will equal 1 (no decimation) and displacements will typically be much larger than 3 pixels. In very low velocity regions, if Δk_i is larger than F, the number of frames in one sweep, it is set to F-1 to enable at least one measurement per sweep.

2.2.2 Sinusoidal Velocity

We define frame decimation vector as:

$$\Delta k(k)_i = \frac{x_{ideal}}{x_{theo}(k)_i}, \qquad \text{(S1)}$$

where $x_{\text{shift}}(k)_i$ is the theoretical pixel displacements over frames k in $\text{ROI}_{i,j}$. The maximum frame decimation number is capped at a user defined number based on a maximum phase angle of the waveform to search over: $\theta_{max} = \frac{2\pi}{10}$, so that the velocity is not changing significantly over the decimation range.

Note that the velocity of a time point must be calculated as the average velocity between two equally distant frames. We perform normalized cross correlations using the decimation

frame pairs of each row and store the x_{shift} values in a master ($N \times M \times Q$) matrix. The velocity is calculated by dividing each x_{shift} by total number of frames between the two time points.

$$U(k)_{i,j} = \frac{x_{shift}(k)_{i,j}}{1 + 2 * \Delta k(k)_{i,j}}. \quad \text{(S2)}$$

The outcome velocities have units of pixels per frame. The results of the decimation technique are displayed in Figure S1.

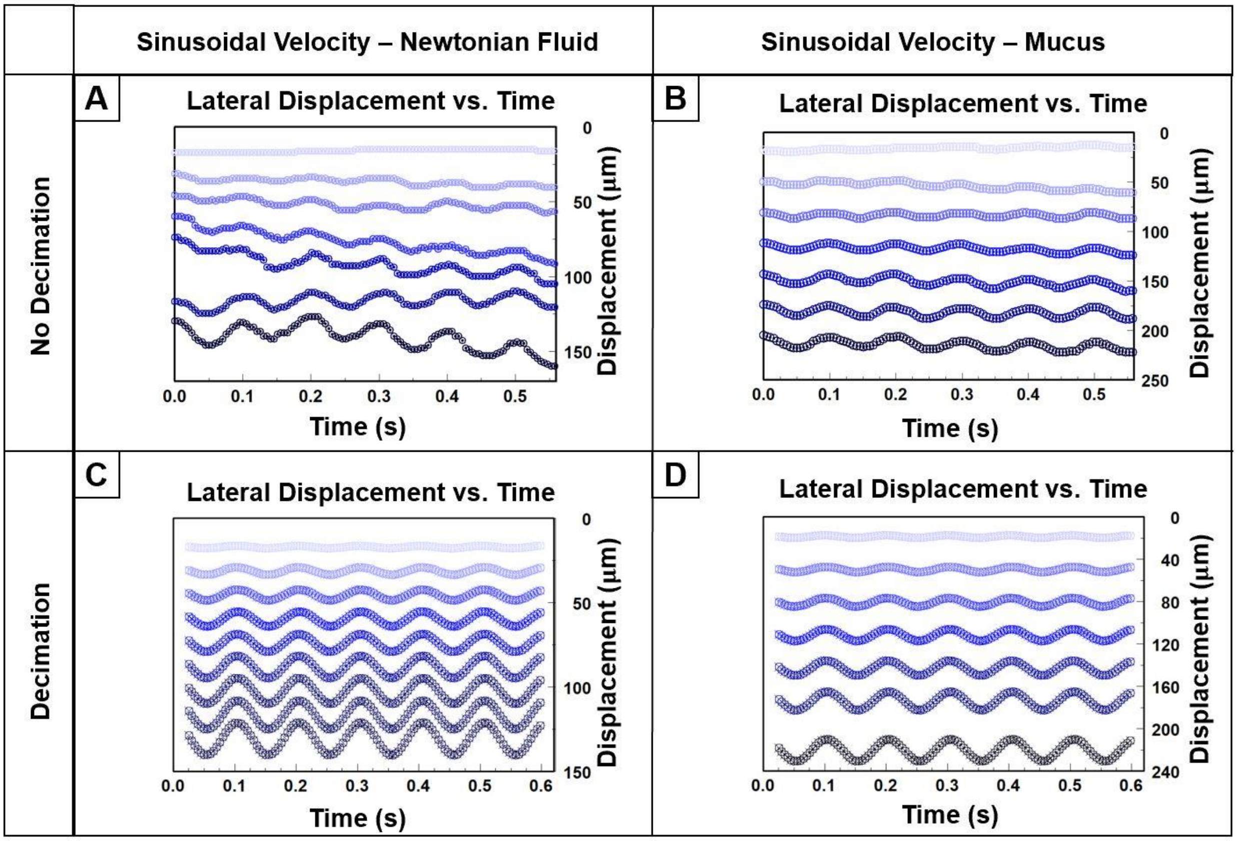

Figure S1: (a) Pre-decimation traces of lateral particle displacements in a Newtonian sample driven by a sinusoidal waveform. (b) Pre- decimation traces of lateral particle displacements in a mucus sample driven by a sinusoidal waveform. **:** (c) Post-decimation traces of lateral particle displacements in a Newtonian sample driven by a sinusoidal waveform. (d) Post-decimation traces of lateral particle displacements in a mucus sample driven by a sinusoidal waveform. Traces are shown at multiple depths in the sample, offset by their depth position for clarity (5 of 6 waveforms shown). For visual purposes, not all ROI depths were plotted.
